# Lower proteinuria is better for patients with IgA nephropathy: a systematic review

**DOI:** 10.3389/fneph.2025.1722582

**Published:** 2026-01-07

**Authors:** Ankit Shah, Manish Maski, Ogo Egbuna, Whitney Longstaff, Janice Stricker-Shaver, Beth Barber

**Affiliations:** 1 Vertex Pharmaceuticals Incorporated, Boston, MA, United States; 2 Maple Health Group, LLC, New York, NY, United States

**Keywords:** IgA, nephropathy, IgAN, proteinuria, kidney failure, systematic review

## Abstract

**Background:**

Proteinuria is a well-established and recommended biomarker for disease activity in patients with IgAN. In the most recent version of the KDIGO guideline, the target level of proteinuria changed from < 1.0 g/day to < 0.5 g/day. The objective of this systematic literature review (SLR) is to identify, synthesize, and critically evaluate the evidence from peer-reviewed publications that inform the significance of achieving different proteinuria levels.

**Methods:**

We searched PubMed and Embase (2005-2025) for studies in adult patients diagnosed with IgAN that examined the relationship between proteinuria measured by any method (e.g., uPCR, 24-hour protein excretion) and key kidney outcomes. The review used an *a priori* protocol following established methodological guidance for systematic reviews. Additionally, the quality of all studies included in the SLR was assessed based on standardized appraisal tools. The evidence was narratively synthesized reporting frequencies and percentages.

**Results:**

Twenty-one unique studies were included (representing 13,006 patients with IgAN). The studies captured in the SLR were mostly observational and they encompassed diverse patient populations, timing of proteinuria assessment, methods of proteinuria measurement and classification, and clinical management strategies, reflecting real-world heterogeneity in IgAN. Despite the differences in individual study methods, results across studies consistently found that lower proteinuria was associated with better kidney outcomes. Specifically, it was clearly established that <0.5 g/day achieved better outcomes than higher proteinuria thresholds.

**Conclusion:**

The evidence identified in this SLR affirms the updated KDIGO recommendation to achieve at least a proteinuria level of < 0.5 g/day.

**Systematic Review Registration:**

https://www.crd.york.ac.uk/prospero/, identifier CRD420251062821.

## Introduction

Immunoglobulin A nephropathy (IgAN) is the most common biopsy-proven primary glomerular disease (1). It can affect individuals of all ages, although patients are typically diagnosed between the ages of 25 to 35 years (2, 3). The underlying mechanism involves an immune-mediated response characterized by deposition of IgA-containing immune complexes in the mesangium, leading to mesangial hypercellularity and inflammation, manifesting clinically as hematuria and proteinuria (4, 5). Prolonged glomerular injury leads to scar formation, initial hyperfiltration, subsequent reduced glomerular filtration rate (GFR), and eventually may progress to chronic kidney disease (CKD) and end-stage kidney disease (ESKD) requiring dialysis or kidney transplantation (6).

Most patients with IgAN are at risk to progress to kidney failure during their lifetime, regardless of age or estimated glomerular filtration rate (eGFR) at diagnosis, with 30% to 40% progressing within 20 years following diagnosis (7–9). Among adult patients with proteinuria > 0.5 g/day or eGFR < 60 mL/min/1.73m^2^ per year, 72% progress to kidney failure/death within 20 years (9). Moreover, median life expectancy is substantially reduced by 10 years (2).

Proteinuria is a well-established and recommended biomarker for disease activity in patients with IgAN. It is also a strong predictor of adverse kidney outcomes, including decline in eGFR, progression to ESKD, need for dialysis, and kidney transplant (10). Higher levels of proteinuria are linked to faster rates of kidney function decline and mortality (10).

The most recent Kidney Disease: Improving Global Outcomes (KDIGO) international guideline (2025) (11) recommends 24-hour proteinuria testing for initial disease staging followed by regular spot urine protein-to-creatinine ratio (UPCR) testing for routine monitoring of disease management and progression. The guidelines put emphasis on three pillars for the treatment of IgAN: (1) use of therapies like renin-angiotensin-aldosterone-system (RAS) and sodium-glucose cotransporter-2 (SGLT2) inhibitors to treat established nephron loss and glomerular hyperfiltration; (2) use of steroids and complement inhibitors to reduce inflammation and immediate consequences of inflammation; and (3) simultaneous use of investigational agents (e.g., BAFF and/or APRIL inhibitors, critical regulators of B cell function) to reduce pathogenic forms of IgA and IgA immune complex formation.

The most recent KDIGO guidelines further recommend maintaining proteinuria levels < 0.5 g/day (or equivalent), preferably < 0.3 g/d (or equivalent), to reduce the rate of loss of kidney function to < 1 ml/min per year for the rest of the patient’s life, as IgAN patients with proteinuria ≥ 0.5 g/day (or equivalent) are at risk of progressive, irreversible loss of kidney function (11). This represents a change from the previous guideline (2021) (12) which recommended bringing proteinuria levels down to < 1.0 g/day. This systematic literature review (SLR) explored the evidence base characterizing the relevance of proteinuria levels on kidney outcomes including kidney survival and dialysis, and the extent to which there are benefits from achieving the target levels of proteinuria specified in the updated KDIGO guideline (11).

## Methods

This SLR was performed in accordance with the methodological principles of conduct for SLRs/literature reviews as detailed in the University of York Centre for Reviews and Dissemination (CRD)’s “Guidance for Undertaking Reviews in Health Care” and reported per the Preferred Reporting Items for Systematic Literature Reviews and Meta-Analyses (PRISMA) statement (2020) (13).

The primary research question was ‘in patients with IgAN, is there a relationship between proteinuria levels and kidney outcomes, including: eGFR, the development of ESKD/end-stage renal disease (ESRD), dialysis, kidney replacement therapy (KRT), and mortality.’ A secondary question was ‘does achieving a specific threshold of proteinuria associate with improved kidney outcomes, such as slower eGFR decline, reduced risk of ESKD, dialysis, kidney transplant, or mortality?’.

### Search strategy

A comprehensive search was conducted in Embase (via Embase.com) and PubMed (NLM) databases (**Supplementary Tables 1**-**3**). The individual search strategies were developed by researching appropriate terms (both subject headings [i.e., MeSH and Emtree terms] and free text words) for inclusion in the literature search strategy. The strategy was validated by comparing the search results to a sample set of published studies that would be expected to be identified. Searches were conducted over an approximate 20-year publication date range (January 1, 2005, to March 20, 2025). The 2005 cutoff was selected as the earliest date to enable identification of clinical trials for the guideline recommended treatments. Only full-text, peer-reviewed publications with proteinuria categories ≤ 1 g/day (or the equivalent) were included; grey literature sources, preprint servers, clinical trial registries (e.g., clinicaltrial.gov) or conferences were not searched. The bibliographies of relevant SLRs were not hand-searched to identify any additional studies of relevance.

### Study selection

Citations identified in the database searches were exported to an EndNote 20 library. Duplicate citations were removed, and unique citations were exported to an online literature review platform (DistillerSR^®^). Each citation was evaluated against the predetermined population, exposure, outcome, and study design (PEOS) criteria (**Supplementary Table 4**) in two screening phases.

### Title/abstract review

Each title and abstract were independently reviewed against the eligibility criteria by two reviewers. At this stage, all citations clearly meeting the eligibility criteria were included, along with those whose applicability to the eligibility criteria was unclear or for which the abstract was not available.

### Full-text review

**Figure 1:**
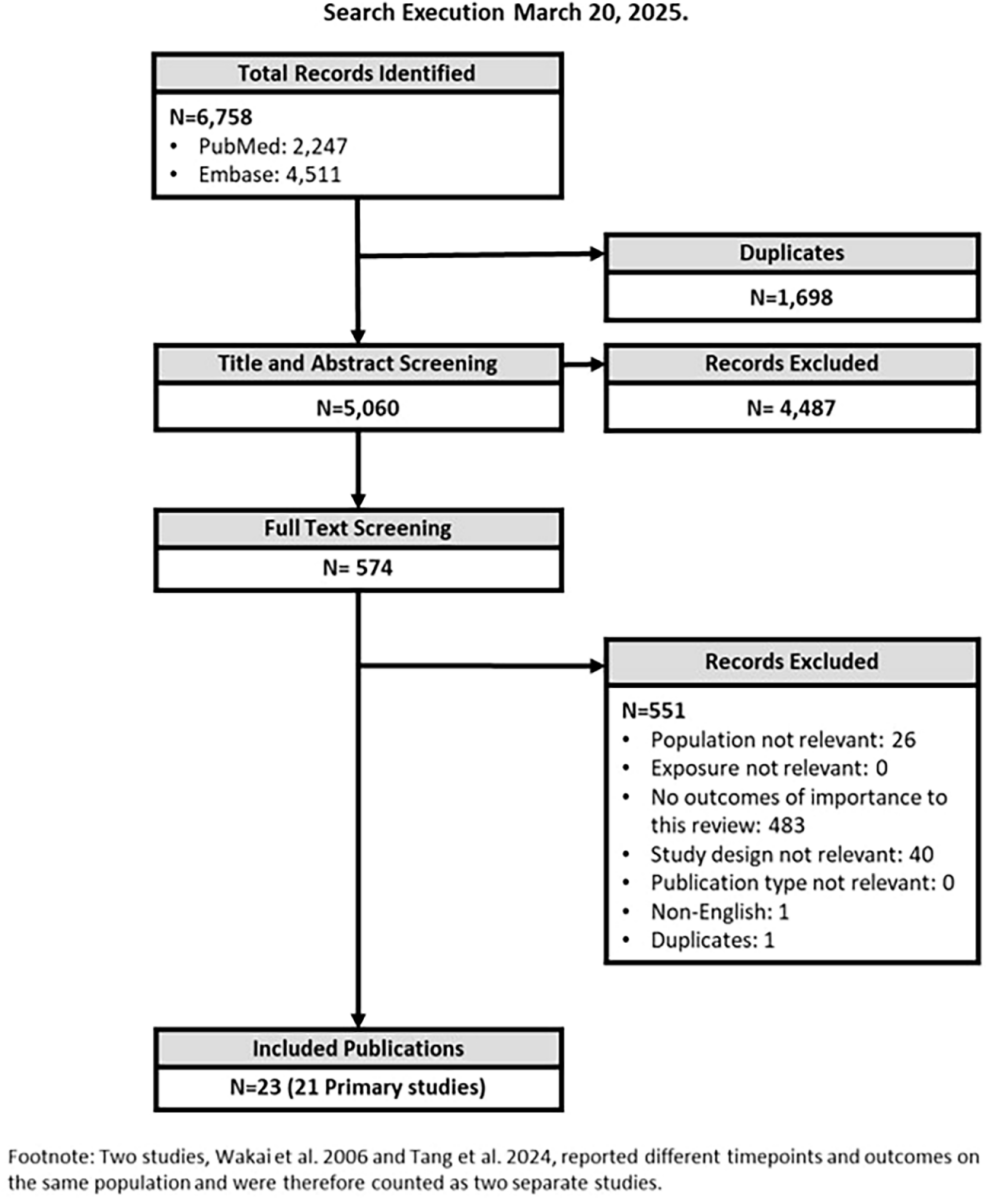
PRIMSA flow diagram.

Full-text publications were independently reviewed against the eligibility criteria by two reviewers. Studies not providing sufficient information for the evaluation of the eligibility criteria were excluded at this stage to ensure that only relevant publications were included. The primary reason for exclusion was captured and recorded in the PRISMA flow diagram (**Figure 1**).

### Data extraction

Data extraction was performed in accordance with the aforementioned guidelines. Data extraction was performed by a single reviewer for each included study and independently verified by a second reviewer once completed. Data were extracted and presented by outcome of interest in an Excel^®^-based data extraction spreadsheet. Information on study design, study location and patient population were extracted, as well as relevant outcomes from the included studies.

### Quality assessment

Quality assessment of the included studies was conducted in accordance with the guidelines from the University of York CRD using the Newcastle Ottawa Tool for real-world cohort studies. The quality assessment across domains (selection, comparability and outcomes) was scored by a single reviewer for each included study and independently verified by a second reviewer once completed. According to the assessment tool, a total score of 0-3, 4–6 and 7–9 represent low, moderate and high quality respectively.

### Conflict resolution

For the title/abstract screening, full-text screening, data extraction and quality assessment phases of the review, any discrepancies or missing information identified by the second reviewer was discussed by both reviewers until a consensus was reached. Any unresolved conflicts were decided by a third senior reviewer.

### Data synthesis

The evidence on the established relationship between proteinuria and key renal endpoints was narratively synthesized and frequencies and percentages were reported.

## Results

### Search results

Of the 6,758 citations identified from the electronic and hand searches, 21 unique studies (9, 10, 14–32) were included (plus two companion publications) (33, 34). These included three prospective cohort studies (17, 23, 29), 17 retrospective cohort studies (9, 10, 14–16, 18–22, 24, 25, 27, 28, 30–32), and one pooled analysis (26) of three studies. The studies included 13,006 patients with IgAN (range: 33 to 2,439 patients (median, 500; interquartile range [IQR], 141 to 1,155) (**Supplementary Table 5**). Studies were published between 2006 and 2025 (median, 2018; IQR, 2015 to 2020). Most studies were conducted in Asian centers. The mean age of patients ranged from 24 to 53 years; 56% of patients were men and all the cohort studies were adjudicated to be of moderate to high quality (**Supplementary Table 7**).

Studies reported median proteinuria as grams per 24 hours (g/day) or urine protein-to-creatinine ratio (uPCR) or urine albumin-to-creatinine ratio (uACR). Treatment of IgAN differed markedly among the included studies (**Supplementary Table 1**), and some studies only classified proteinuria after a period of treatment (e.g., persistent proteinuria). Most studies reported mean eGFR ranging from 33 to 112 mL/min/1.73m^2^ per year; average 75 mL/min/1.73m^2^ per year.

The results from the 21 studies are presented here by the outcomes that were evaluated (e.g. eGFR decline/hazard ratios [HRs] for kidney failure, dialysis and transplant). The mean duration of follow up across all studies was 5.9 years (range: 1.6–14 years). The totality of evidence consistently demonstrates that lower levels of proteinuria result in better kidney outcomes. Of note, there were differences in methods including timing of measurements and definitions of outcomes, therefore, comparisons of estimates across studies should not be made. The majority of the studies analyzed the association between proteinuria and kidney outcomes using Kaplan Meier curves and Cox Proportional Hazards regression analyses (univariate and/or multivariate). The most frequent variables used for covariate adjustment were age, blood pressure or hypertension, eGFR, serum protein or albumin, baseline proteinuria, gender, diabetes and BMI. Five studies didn’t report their adjustment methods.

A PRISMA flow diagram of studies identified and screened for inclusion in this review is provided in **Figure 1**.

### Estimated glomerular filtration rate

Multiple studies reported on the association of proteinuria and change in the eGFR slope (mL/min/1.73m^2^ per year) (**Table 1**). All studies reported worse outcomes (e.g., a steeper eGFR decline) with higher baseline or time-averaged proteinuria (TA-P) levels. Reported eGFR declines were steeper as proteinuria levels increased, and specifically proteinuria categories > 0.5 g/day (or equivalent) had larger eGFR declines than proteinuria categories < 0.5 g/day (or equivalent). Furthermore, Faucon et al., 2025 (16) and Shen et al., 2025 (28) examined more refined category levels for proteinuria. Both studies found that the proteinuria category between 0.51 to 1 g/day (or equivalent) had steeper eGFR decline than the proteinuria category < 0.5 g/day (or equivalent) (16, 28). Specifically, Shen et al., 2025 reported the rate of eGFR decline was significantly more rapid in the 0.5–1.0 g/day [Mean -1.6 (95% CI -1.7 to -1.5)] and ≥ 1.0 g/day [Mean -3.3 (95% CI -3.4 to -3.2)] groups than the 0.3–0.5 g/day group [Mean -0.8 (95% CI -0.9 to -0.6)] (28). In addition to the studies in the table, Sevillano et al., 2017 reported that patients with proteinuria ≤ 0.75 g/day had a significantly lower kidney function decline (-1.08 *vs*. -4.20; p=0.02) compared to patients with proteinuria > 0.75 g/day (27).

**Table 1:** Relationship between proteinuria (uPCR/uACR/24-hr urine protein) and annual eGFR slope.

Study	Annual eGFR slope by proteinuria categories (uACR/uPCR: g/g and 24-hr urine protein: g/d)						
	≤ 0.30	0.31–0.50	0.51–1.00	1.00–1.50	1.50–2.00	2.00–2.99	≥ 3.00
Faucon 2025 (16) (uACR)	Mean: -0.74 (95% CI: -1.18 to -0.29)	Mean: -2.07 (95% CI: -2.82 to -1.32)	Mean: -3.35 (95% CI: -3.91 to -2.79)	Mean: -4.28 (95% CI: -5.04 to -3.51)	Mean: -6.07 (95% CI: -7.07 to -5.06)	Mean: -6.52 (95% CI: -7.26 to -5.78)	
Stamellou 2024 (29) (uACR)	Mean: -1.49 (95% CI: NR)		Mean: -2.34 (95% CI: NR)		Mean: -3.16 (95% CI: NR)		
Nam 2014 (25) (uPCR)	Mean: -0.41 (SD: 1.68)	Mean: -0.73 (SD: 2.82)		Mean: -4.07 (SD: 6.54)			Mean: -9.35 (SD: 13.1)
Shen 2025 (28) (Urine protein)	Mean: -0.5 (95% CI: -0.7 to -0.4)	Mean: -0.8 (95% CI: -0.9 to -0.6)	Mean: -1.6 (95% CI: -1.7 to -1.5)	Mean: -3.3 (95% CI: -3.4 to -3.2)			
Pitcher 2023* (10) (uPCR)	Mean: -0.0 (SD: 7.3)		Mean: -1.1 (SD: 5.7)	Mean: -3.8 (SD: 5.5)		Mean: -9.5 (SD: 9.4)	

*uPCR categories were converted into urine protein categories.

CI, confidence intervals; NR, not reported; SD, standard deviation, eGFR, estimated glomerular filtration rate; g/g, grams of protein per gram of creatinine; g/d, grams of protein per day; uACR, urine albumin-to-creatinine ratio, uPCR, urine protein-to-creatinine ratio.

### Kidney failure

Eighteen studies reported on the association between proteinuria and kidney failure (**Table 2** and **Supplementary Table 6**). Kidney failure was defined differently among these included studies (e.g., the use of serum creatinine doubling, eGFR decline, the use of KRT, and/or transplantation). Roughly 50% of the included studies defined kidney failure as a composite outcome (e.g., Composite Kidney Endpoint [CKE] or Major Adverse Kidney Events [MAKE]) with varying definitions of the events that would constitute the endpoint. Across these studies, the rate of kidney damage was associated with the level of baseline or TA-P levels. Despite definitional variations, all studies reported worse outcomes (e.g., worse kidney survival) with higher proteinuria levels (baseline or time-averaged) (35).

**Table 2:** Relationship between proteinuria (uPCR/uACR/24-hr urine protein) and kidney failure.

Study	Kidney failure	Proteinuria categories (uACR/uPCR: g/g and 24-hr urine protein: g/d)						
		≤ 0.30	0.31–0.50	0.51–1.00	1.00–1.50	1.50–2.00	2.00–2.99	≥ 3.00
Ai 2020 (14) (urine protein)	SCr doubling	Ref	HR 3.70 (95% CI 1.09 to 12.56)	HR 3.67 (95% CI 1.23 to 10.94)	HR 8.2 (95% CI 2.89 to 23.26)			
	eGFR <15*, dialysis, transplantation	Ref	HR 2.72 (95% CI 0.74 to 9.98)	HR 2.12 (95% CI 0.67 to 6.70)	HR 6.04 (95% CI 2.08 to 17.5)			
Shen 2025 (28) (urine protein)	eGFR < 15*, KRT, transplantation	Ref	HR 1.08 (95% CI 0.43 to 2.70)	HR 4.61 (95% CI 2.37 to 8.96)	HR 15.61 (95% CI 8.14 to 29.93)			
Le 2012 (9) (urine protein)	eGFR <15*, dialysis, transplantation, eGFR decline >50%	Ref		HR 9.10 (95% CI 2.70 to 30.00)	HR 46.50 (95% CI 14.70 to 147.50)			
Faucon 2025 (16) (uACR)	eGFR decline >30% and KRT	Ref	HR 1.56 (95% CI 1.14 to 2.14)	HR 2.04 (95% CI 1.58 to 2.64)	HR 2.82 (95% CI 2.10 to 3.78)	HR 4.21 (95% CI 3.08 to 5.74)	HR 4.53 (95% CI 3.36 to 6.11)	
	eGFR decline >30%	Ref	HR 1.76 (95% CI 1.26 to 2.47)	HR 2.20 (95% CI 1.66 to 2.91)	HR 2.45 (95% CI 1.78 to 3.39)	HR 3.20 95% CI 2.25 to 4.56)	HR 3.47 (95% CI 2.47 to 4.87)	
	KRT	Ref	HR 1.39 (95% CI 0.94 to 2.03)	HR 1.84 (95% CI 1.35 to 2.51)	HR 2.59 (95% CI 1.84 to 3.64)	HR 4.14 (95% CI 2.89 to 5.91)	HR 4.65 (95% CI 3.28 to 6.59)	
Nam 2014 (25) (uPCR)	eGFR decline >50%	Ref	HR 2.82 (95% CI 0.32 to 24.72)		HR 25.00 (95% CI 3.17 to 197.00)			
Tang 2024 (33) (urine protein)	ESKD, eGFR decline >50%	Ref	HR 2.22 (95% CI 0.88 to 5.58)	HR 4.04 (95% CI 1.93 to 8.46)	HR 8.46 (95% CI 3.80 to 18.83)		HR 38.00 (95% CI 17.62 to 81.95)	
Takada 2019 (31) (uPCR)	eGFR decline >30%	Ref		HR 0.80 (95% CI 0.30 to 2.08)	HR 2.23 (95% CI 0.91 to 5.48)			
Goto 2009 (17) (urine protein)	Dialysis	Ref	HR 3.41 (95% CI 1.29 to 9.06)					
Tanaka 2013 (32) (urine protein/uPCR)	KRT	Ref		HR 3.32 (95% CI 0.69 to 15.9)				
Gutierrez 2012 (18) (urine protein)	>50% SCr increase	Ref		HR 5.30 (95% CI 0.84 to 33.36)				
Koike 2024 (23) (urine protein)	>50% baseline SCr increase, dialysis	Ref		HR 3.55 (95% CI 1.61 to 7.83)				
	>50% baseline SCr increase, dialysis	Ref		HR 3.91 (95% CI 1.78 to 8.56)				
Chen 2018 (15) (urine protein)	>50% SCr increase				Ref	HR 2.43 (95% CI 1.36 to 4.36)		
Stangou 2018 (30) (urine protein)	SCr doubling	Ref		HR 2.38 (95% CI: 1.18 to 4.82)				
	ESKD [no further definition provided]	Ref		HR 7.14 (95% CI: 1.61 to 31.76)				
Hirano 2013 (19) (urine protein)	>50% SCr increase	HR 0.06 (95% CI 0.01 to 0.57)	HR 0.24 (95% CI 0.04 to 1.25)		Ref			

*mL/min/1.73m^2^ per year.

%, percent; eGFR, estimated glomerular filtration rate; ESKD, end-stage kidney disease; ESRD, end-stage renal disease; KRT, kidney replacement therapy; Ref, reference; SCr, serum creatinine, CI, confidence intervals; g/g, grams of protein per gram of creatinine; g/d, grams of protein per day; HR, hazard ratio; uACR, urine albumin-to-creatinine ratio, uPCR, urine protein-to-creatinine ratio.

Specifically, Le et al., 2012 (9) reported that patients with proteinuria > 1.0 g/day were at an increased risk for the composite endpoint (50% reduction in kidney function or ESKD) *vs* those with proteinuria < 0.5 g/day (HR 46.5, 95% CI 14.7 to 147.5, p < 0.001). This increased risk for the composite endpoint was also demonstrated for patients with proteinuria 0.5–1.0 g/day *vs* proteinuria < 0.5 g/day (HR 9.1, 95% CI 2.7 to 30.0, p<0.001).

**Figure 2:**
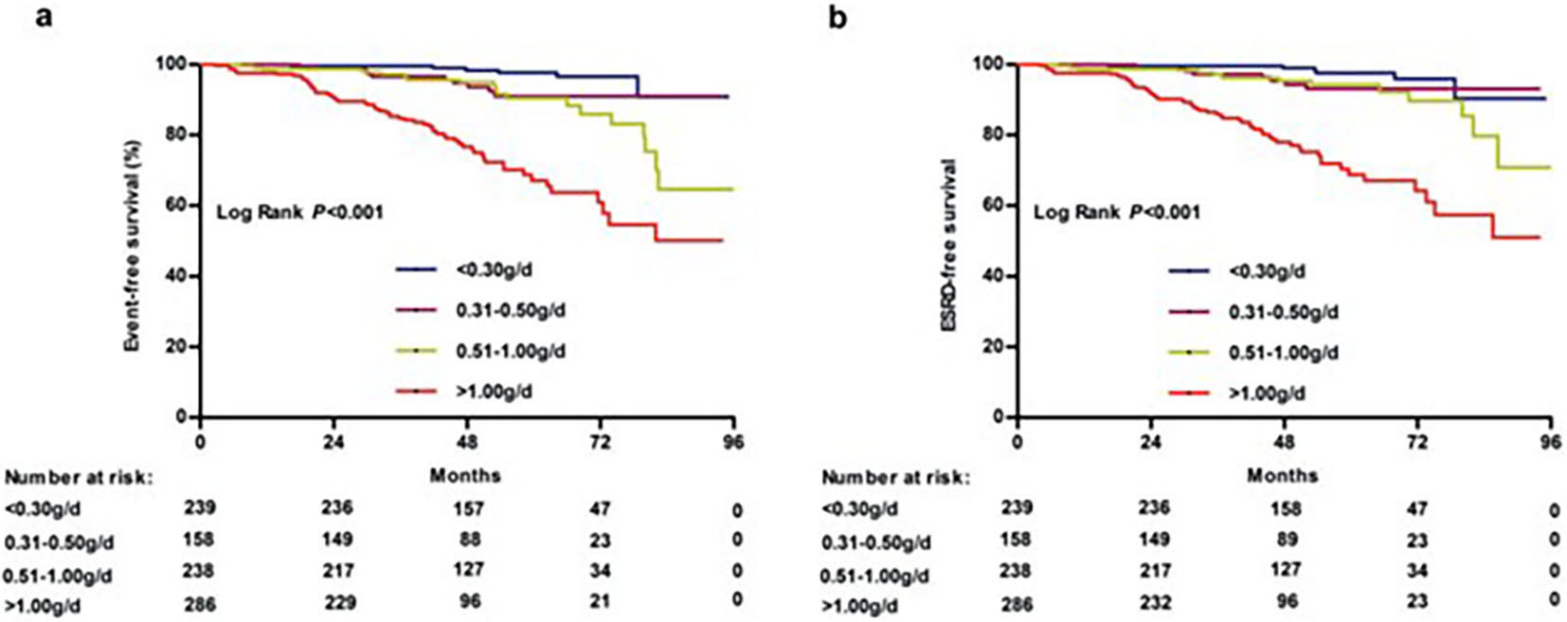
Renal survival curve for patients presenting with different levels of proteinuria: **(a)** calculated from doubling of baseline serum creatinine; **(b)** calculated from end-stage renal disease (14).

**Figure 3:**
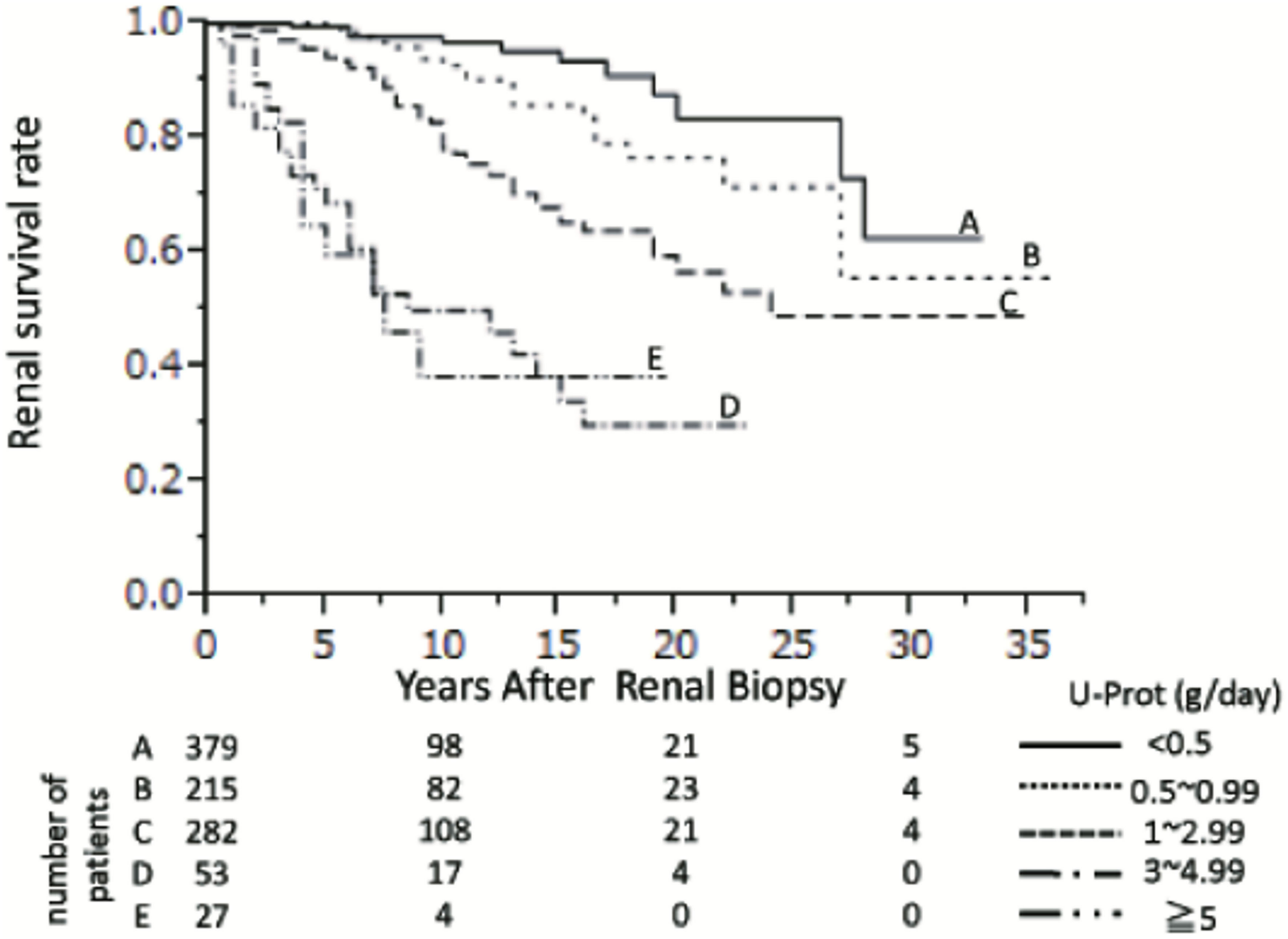
Cumulative kidney survival rates in patients with IgAN categorized by urine proteinuria (24).

**Figure 4:**
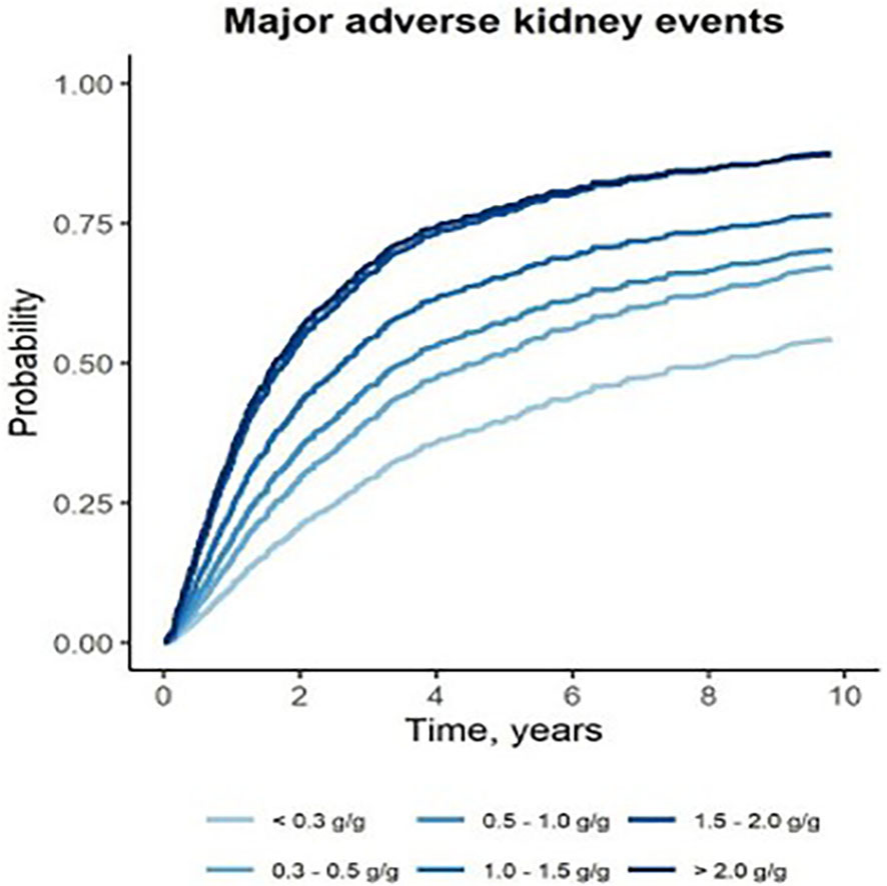
Adjusted cumulative incidence for major kidney disease event (kidney replacement therapy or eGFR decline > 30%) by baseline proteinuria (16).

Also of importance, four studies (Ai et al., 2020, Faucon et al., 2025, Shen et al., 2025, Tang et al., 2024) (14, 16, 28, 33) that compared proteinuria levels < 0.30 to 0.31–0.50 g/day (or equivalent) found that achieving proteinuria levels < 0.3 g/day (or equivalent) had a significant benefit on kidney survival. For example, Ai et al., 2020 (14) reported that the kidney survival rate (as calculated by doubling of serum creatinine levels) were significantly lower in patients with proteinuria0.31–0.50 g/day compared to ≤ 0.30 g/day (p < 0.04). Moreover, after adjustment for known risk factors, there was a significant prognostic effect of proteinuria < 0.3 *vs*. 0.31–0.50 g/day (HR 3.70, 95% CI 1.09 to 12.56). A number of studies also included figures that highlighted the differences in time to kidney failure by proteinuria category; displayed are some examples (**Figures 2**-**4**). These figures all included the proteinuria category < 0.5 g/day (or equivalent) and all of these studies demonstrated reduced risk of kidney failure for the proteinuria category < 0.5 g/day (or equivalent) *vs* 0.5–1 g/day (or equivalent). In the studies that evaluated time to kidney failure (**Figures 2** and **3**), separation in the curves for proteinuria categories < 0.5 g/day (or equivalent) *vs* 0.5–1.0 g/day (or equivalent) began to occur around the 6–7-year timepoint whereas the separation occurred much earlier for higher proteinuria categories. Faucon et al., 2025 (16) evaluated the cumulative incidence rates for MAKE by baseline proteinuria levels and the curves for < 0.5 g/day (or equivalent) *vs* 0.5–1.0 g/day (or equivalent) began to separate before the 2-year timepoint.

**Figure 5:**
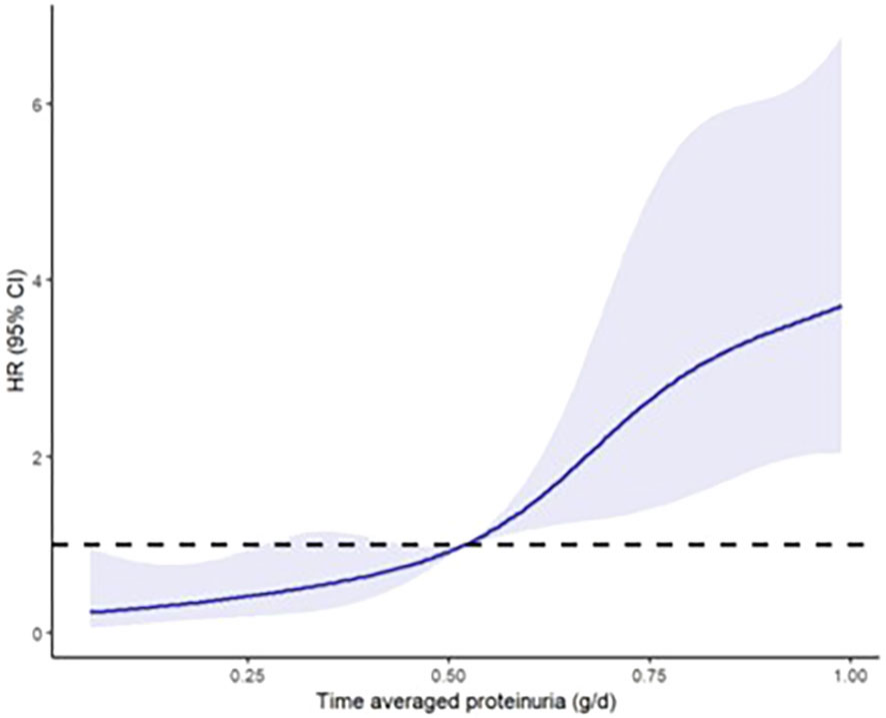
Restricted cubic splines to model the nonlinear relationship between time-averaged proteinuria and the risk of end-stage kidney disease (28).

**Figure 5** displays the restricted cubic splines modeling the nonlinear relationship between TA-P and the risk of ESKD (28). Of note, when proteinuria levels rose above 0.5 g/day the risk for ESKD increased significantly.

### Serum creatinine increase from baseline

Five studies reported on the incidence of serum creatinine increase from baseline (**Table 3**) (14, 15, 18, 19, 30). All the studies reported that lower proteinuria levels were associated with a lower incidence and lower risk of serum creatinine increase from baseline.

**Table 3:** Relationship between proteinuria (uPCR/uACR/24-hr urine protein) and serum creatinine (50% increase or doubling from baseline).

Study	Kidney failure	Proteinuria (uACR/uPCR: g/g and 24-hr urine protein: g/d)			
		≤ 0.30	0.31–0.50	0.51–1.00	≥ 1.00
Chen 2018 (15) (urine protein)	>50% SCr increase	Ref		HR 2.43 (95% CI 1.36 to 4.36)	
Gutierrez 2012 (18) (urine protein)	>50% SCr increase	Ref		HR 5.30 (95% CI 0.84 to 33.36)	
Hirano 2013 (19) (urine protein)	>50% SCr increase	HR 0.06 (95% CI 0.01 to 0.57)	HR 0.24 (95% CI 0.04 to 1.25)		Ref
Ai 2020 (14) (uPCR)	SCr doubling	Ref	HR 3.70 (95% CI 1.09 to 12.56)	HR 3.67 (95% CI 1.23 to 10.94)	HR 8.20 (95% CI 2.89 to 23.26)
Stangou 2018 (30) (urine protein)	SCr doubling	Ref		HR 2.38 (95% CI: 1.18 to 4.81)	

%, percent; HR, hazard ratio; Ref, reference; CI, confidence intervals; g/g, grams of protein per gram of creatinine; g/d, grams of protein per day; KRT, kidney replacement therapy; SCr, serum creatinine; uACR, urine albumin-to-creatinine ratio, uPCR, urine protein-to-creatinine ratio.

### KRT and dialysis

Four studies reported on the need for KRT/dialysis based on baseline proteinuria (**Table 4**) (16, 17, 32, 34). All studies reported that lower proteinuria levels were associated with a lower risk of KRT or dialysis.

**Table 4:** Relationship between proteinuria (uPCR/uACR/24-hr urine protein) and kidney replacement therapy/dialysis.

Study	Kidney failure	Proteinuria (uACR/uPCR: g/g and 24-hr urine protein: g/d)						
		≤ 0.30	0.31–0.50	0.51–1.00	1.00–1.50	1.50–2.00	2.00–2.99	≥ 3.00
Faucon 2025 (16) (uACR)	KRT	Ref	HR 1.39 (95% CI 0.94 to 2.03)	HR 1.84 (95% CI 1.35 to 2.51)	HR 2.59 (95% CI 1.84 to 3.64)	HR 4.14 (95% CI 2.89 to 5.91)	HR 4.65 (95% CI 3.28 to 6.59)	
Tanaka 2013 (32) (urine protein)	KRT	Ref		HR 3.32 (95% CI 0.69 to15.9)				
Goto 2009 (17) (urine protein)	Dialysis	Ref	HR 3.41 (95% CI 1.29 to 9.06)					
Wakai 2006 (34) (urine protein)	Dialysis	Ref	RR 2.97 (95% CI 0.86 to 10.30)					

%, percent; HR, hazard ratio; RR, relative risk; Ref, reference.

## Discussion

This SLR explored the evidence base characterizing the significance of proteinuria levels on the risk of key kidney outcomes in IgAN, and the extent to which there are benefits from achieving the target levels of proteinuria specified in the updated KDIGO guideline. Twenty-one primary studies were identified that analyzed data on a total of 13,006 patients with IgAN. There were differences across the studies, including methodologies employed, IgAN patient populations selected and the timing of proteinuria assessment. Additionally, a range of kidney outcomes were studied, including decline in eGFR, progression to ESRD or ESKD, initiation of dialysis, and various composite endpoints such as MAKE and clinically significant kidney events.

Despite variation in the kidney outcomes measured, study methods, patient populations, treatment of IgAN, and timing of proteinuria assessment, a consistent pattern emerged: lower levels of proteinuria were associated with more favorable kidney outcomes, and in particular, achieving a proteinuria < 0.5 g/day (or equivalent) was consistently associated with better outcomes than higher categories, including values in the range of 0.5–1.0 g/day. Additionally, Ai et al., 2020 (14), Faucon et al., 2025 (16), Shen et al., 2025 (28) and Tang et al., 2024 (33) that compared proteinuria levels <0.3 to 0.31–0.5 g/day found significant benefit on kidney outcomes by achieving proteinuria levels < 0.3 g/day.

These findings are aligned with the most recent KDIGO guideline (2025) (11), which advocates for the reduction of proteinuria as a key therapeutic target in the management of IgAN and recommends target proteinuria levels < 0.5 g/day. An older KDIGO guideline (2021) (12) recommended target proteinuria levels < 1.0 g/day, but the findings of this SLR have shown that there is significant benefit achieving proteinuria levels < 0.5 g/day *vs*. between 0.5–1.0 g/day or higher. A recent study by Sim et al., 2025 (36), that was not captured in this SLR due to the March 20^th^, 2055 cutoff, also reported findings consistent with this SLR. Sim et al., 2025 (36) reported that proteinuria levels ≥ 0.5 g/g were associated with increased risk of kidney failure (based on the composite outcome of ≥ 50% eGFR decline, kidney failure, or mortality).

The strengths of this review include the comprehensiveness of the literature search, which encompassed two major medical databases, and the use of an *a priori* protocol following established methodological guidance for systematic reviews. Additionally, all studies included in the SLR were assessed as being of high quality based on standardized appraisal tools, which strengthened the reliability of the findings. However, this review also has limitations. The studies captured in the SLR are mostly observational and they encompassed diverse patient populations, reflecting real-world heterogeneity in IgAN. There was also variation in the timing of proteinuria assessment (e.g., at the time of kidney biopsy versus following a period of treatment), methods of proteinuria measurement and classification, and clinical management strategies. These factors may introduce heterogeneity that limits the comparability of exact estimates across studies. Furthermore, most studies identified in this SLR were conducted in Asia (14 in Asia and 7 in EU/UK) which may limit generalizability of findings to other populations.

## Conclusions

This systematic review in patients with IgAN highlights the consistent association between lower levels of proteinuria and improvements in a wide range of long-term kidney outcomes, including kidney failure, defined in the following ways: serum creatinine doubling, eGFR decline, the use of KRT, and/or transplantation and several composite outcomes such as CKE and MAKE. The evidence identified in this SLR affirms the KDIGO recommendation to achieve at least a proteinuria level of < 0.5 g/day, supporting its use as a clinically meaningful treatment target. These findings reinforce the importance of proteinuria reduction as both a prognostic marker and therapeutic goal in the management of IgAN and underscore the need for standardized reporting in future studies to enable cross-study comparison of estimates. Given that kidney scarring and function loss are irreversible, these results also reinforce the urgency to reach and maintain the proteinuria target level of < 0.5 g/day early in the course of the disease.

## Data Availability

The original contributions presented in the study are included in the article/**Supplementary Material**. Further inquiries can be directed to the corresponding author.
