# Supplementary material for: Lower proteinuria is better for patients with IgA nephropathy: a systematic review
Source: Front Nephrol. 2026 Jan 7;5:1722582. doi: 10.3389/fneph.2025.1722582 (PMC12819803; doi:10.3389/fneph.2025.1722582)
Supplement: Supplementary file 1 [file DataSheet1.docx]

Supplementary Material

**Supplemental Material Table of Contents**

[Supplemental Table 1. Summary of searches executed on March 20, 2025 2](#_Toc214292613)

[Supplemental Table 2. PubMed search strategy 3](#_Toc214292614)

[Supplemental Table 3. Embase (via Embase.com) search strategy 4](#_Toc214292615)

[Supplemental Table 4. PEOS Criteria 5](#_Toc214292616)

[Supplemental Table 5. Study designs and patient characteristics of the included studies 6](#_Toc214292617)

[Supplemental Table 6. Risk ratio of renal failure based on baseline proteinuria 9](#_Toc214292618)

[Supplemental Table 7. Quality assessment according to the Newcastle–Ottawa Scale 10](#_Toc214292619)

Electronic Database Search Strategy

Supplemental Table 1. Summary of searches executed on March 20, 2025

|  | **Hits** |
| --- | --- |
| PubMed | 2,247 |
| Embase | 4,511 |
| Total | 6,758 |
| Duplicates | 1,698 |
| **Total after de-duplication** | 5,060 |

Supplemental Table 2. PubMed search strategy

| **Domain** | **No.** | **Search terms** | **Hits** |
| --- | --- | --- | --- |
| **IgAN** | 1 | "glomerulonephritis, iga"[MeSH] | 7,841 |
|  | 2 | "glomerulonephritis"[MeSH:noexp] AND "immunoglobulin a"[MeSH] | 791 |
|  | 3 | "IgA" AND "nephropath*" | 10,272 |
|  | 4 | "iga glomerulonephritis"[tiab: ~3] OR "igan" | 5,220 |
|  | 5 | ("immunoglobulin a" OR "immunoglobulin IgA"[tiab: ~3]) AND ("nephropath*" OR "glomerulonephritis") | 4,760 |
|  | 6 | "Bergers disease" OR "Berger's disease" OR "Berger disease" | 230 |
|  | 7 | #1 OR #2 OR #3 OR #4 OR #5 OR #6 | 12,623 |
| **Proteinuria** | 8 | proteinuria OR "creatinine/urine"[Mesh] OR "protein creatinine"[tiab: ~3] OR "protein creatinin"[tiab: ~3] OR "proteinuria creatinuria"[tiab: ~3] OR "UPCR" OR "PCR" OR "P/C ratio" OR "albumin creatinin"[tiab: ~3] OR "albumin creatinine"[tiab: ~3] OR "UACR" OR "ACR" OR “albuminuria” OR “albumin urine”[tiab: ~3] OR "protein excretion"[tiab] OR "UPE"[tiab] | 789,867 |
| **IgAN and proteinuria** | 9 | #7 AND #8 | 4,389 |
| **Animal studies** | 10 | ("Animals"[Mesh] NOT "Humans"[Mesh]) OR "animal"[ti] OR "mouse"[ti] OR "mice"[ti] OR "swine"[ti] OR "porcine"[ti] OR "murine"[ti] OR "sheep"[ti] OR "lambs"[ti] OR "pigs"[ti] OR "piglets"[ti] OR "rabbit"[ti] OR "rabbits"[ti] OR "cat"[ti] OR "cats"[ti] OR "dog"[ti] OR "dogs"[ti] OR "cattle"[ti] OR "bovine"[ti] OR "monkey"[ti] OR "monkeys"[ti] OR "trout"[ti] OR "marmoset"[ti] OR "in vitro"[ti] OR "in vivo"[ti] | 6,000,899 |
| **Irrelevant study types** | 11 | "Letter"[pt] OR "Comment"[pt] OR "Editorial"[pt] OR "Case Reports" [pt] | 4,567,117 |
| **Exclusions** | 12 | #10 OR #11 | 10,430,404 |
| **IgAN and proteinuria after exclusions** | 13 | #9 NOT #12 | 3,462 |
| **English language** | 14 | #13 AND English[language] | 3,128 |
| **Published between 2005 and 2025** | 15 | #14 AND ("2005/01/01"[dp]:"2025/03/20"[dp]) | 2,247 |
| **TOTAL:** | | | 2,247 |

Supplemental Table 3. Embase (via Embase.com) search strategy

| **Domain** | **No.** | **Search terms** | **Hits** |
| --- | --- | --- | --- |
| **Population** | 1 | 'immunoglobulin a nephropathy'/exp | 18,382 |
|  | 2 | 'glomerulonephritis'/de AND 'immunoglobulin a'/exp | 1,902 |
|  | 3 | 'iga' AND 'nephropath*' | 16,002 |
|  | 4 | ('iga' NEAR/3 'glomerulonephritis') OR 'igan' | 8,098 |
|  | 5 | ('immunoglobulin a' OR ('immunoglobulin' NEAR/3 'iga')) AND ('nephropath*' OR 'glomerulonephritis') | 21,783 |
|  | 6 | 'berger$ disease' | 42 |
|  | 7 | #1 OR #2 OR #3 OR #4 OR #5 OR #6 | 23,136 |
| **Proteinuria** | 8 | 'proteinuria'/exp OR 'proteinuria' OR 'protein creatinine ratio'/exp OR ('protein' NEAR/3 'creatinin$') OR ('proteinuria' NEAR/3 'creatinuria') OR 'upcr' OR 'pcr' OR 'p/c ratio' OR 'albuminuria' OR ('albumin' NEAR/3 'urine') OR 'protein excretion' OR 'upe' | 1,204,492 |
| **IgAN and proteinuria** | 9 | #7 AND #8 | 10,189 |
| **Animal studies** | 10 | 'animal'/exp NOT 'human'/exp OR 'animal':ti OR 'mouse':ti OR 'mice':ti OR 'swine':ti OR 'porcine':ti OR 'murine':ti OR 'sheep':ti OR 'lambs':ti OR 'pigs':ti OR 'piglets':ti OR 'rabbit':ti OR 'rabbits':ti OR 'cat':ti OR 'cats':ti OR 'dog':ti OR 'dogs':ti OR 'cattle':ti OR 'bovine':ti OR 'monkey':ti OR 'monkeys':ti OR 'trout':ti OR 'marmoset':ti OR 'in vitro':ti OR 'in vivo':ti | 7,050,940 |
| **Irrelevant study types** | 11 | 'editorial':it OR 'letter':it OR 'note':it | 3,203,075 |
| **Exclusions** | 12 | #10 OR #11 | 10,160,905 |
| **IgAN and proteinuria after exclusions** | 13 | #9 NOT #12 | 9,326 |
| **Conference abstracts** | 14 | #13 AND ('conference abstract':it OR 'conference paper':it OR 'conference review':it) | 3,171 |
| **Full-text articles from 2005 to 2025** | 15 | (#13 NOT #14) AND [2005-2025]/py | 4,744 |
| **English language** | 16 | #15 AND [english]/lim | 4,511 |
| **TOTAL – Full-text articles (2005–2025):** | | | **4,511** |

Supplemental Table 4. PEOS Criteria

| Criteria | Inclusion | Exclusion |
| --- | --- | --- |
| Population | - Adult (≥18 years) patients diagnosed with IgAN regardless of comorbidities | - Non-IgAN populations - Pediatric patients (<18 years) with IgAN |
| Exposure | - Proteinuria reported at two or more different levels below 1.0 g/day or equivalent | - Exposure other than proteinuria |
| Outcomes | - Primary numerical data quantifying the relationship between proteinuria measured by any method (e.g., uPCR, 24-hour protein excretion) and at least one of the following endpoints: - eGFR - ESKD/ESRD development - Dialysis - Kidney transplant - Mortality | - Outcomes other than those described in the inclusion criteria |
| Study Design | - Clinical trials (including post-hoc analyses) - Observational/real-world evidence studies (prospective cohort and cross-sectional studies, retrospective analyses of real-world databases, disease registries, case-control studies) | - Commentaries, letters, notes, editorials, expert opinions, erratums, government reports, reports from specialist societies - Case studies, case reports, case series - Pre-clinical and animal studies - Narrative reviews - Clinical guidelines - SLRs and/or meta-analyses |
| Publication Type | - Peer-reviewed publications | - Non-peer-reviewed publications - Conference abstracts |
| Limits | - Language: English - Full-text articles published in the last 20 years: January 1, 2005 to March 20, 2025 | - Language: Non-English - Full-text articles published in 2004 or before |

Abbreviations: eGFR, estimated glomerular filtration rate; ESKD, end-stage kidney disease; ESRD, end-stage renal disease; IgAN, Immunoglobulin type A nephropathy; PEOS, population, exposure, outcome, and study design; SLR, systematic literature review; uPCR, urine protein-creatinine ratio.

Supplemental Table 5. Study designs and patient characteristics of the included studies

| Study  Study design (Patients)  Study period  Study location | Race/ ethnicity*  Participant age  Percentage Males | Baseline eGFR/  Baseline proteinuria | Treatments received at baseline or before study initiation |
| --- | --- | --- | --- |
| Ai 2020  Retrospective cohort (921)  01/01/2006 – 12/31/2011  China | Chinese patients  Median 32.0  (IQR: 26.0, 38.0)  42.2% males | Median 95.6  (IQR: 61.1, 123.1)/  UP: Median 0.58 g/d  (IQR: 0.30, 1.19) | RAS blockers (80.4%), Corticosteroids (30.4%) |
| Chen 2018  Retrospective cohort (506)  01/1995 - 12/2014  China | Chinese patients  Mean 34.7  (SD 9.5)  54.5% males | Mean 102.1 (SD 19.8)/  UP: 0.56 g/d  (SD 0.26) | ACEI/ARB (79.8%), Steroids (13.6%) |
| Faucon 2025  Retrospective cohort (1269)  01/01/2005 - 12/31/2021  Sweden | Caucasian patients  Median 53  (IQR: 41.0, 66.0)  74.1% males | Mean 33.0 (SD 19.8)/  uACR: Median 0.70 g/g  (IQR: 0.20, 1.50] | RASi (83.1%), Calcium channel blockers (54.0%), Diuretics (43.7%), Beta-blockers (43.4%), Corticosteroids (19.1%), Oral immunosuppressive therapy (4.2%), Antiplatelets (11.7%), Lipid-lowering therapy (45.2%) |
| Goto 2009**  Cross-sectional (surveys) (2283)  1995 - 2005  Japan | Japanese patients  Median 32.1  (IQR: 20.7, 46.9)  48.7% males | NR/ NR | Corticosteroids (34.5%), immunosuppressive agents (10.6%), angiotensin-converting enzyme inhibitors (28.2%) |
| Gutierrez 2012  Retrospective cohort (141)  1975 - 2008  Spain | Caucasian patients  Mean 23.7  (SD 14.8)  63.8% males | Mean 111.7 (SD 31.6)/  UP: Median 0.20 g/d  (IQR: 0.10, 0.40) | Renin-angiotensin system (RAS) blockers (41.8%) |
| Hirano 2013  Retrospective cohort (141)  2004 - 2010  Japan | Japanese patients  Median 34.0  (IQR: 26.0, 43.0)  48.9% males | Mean 72.8 (SD 28.0)/  UP: Median 1.00 g/d  (IQR: 0.65, 1.70) | Tonsillectomy (48.2%), RAAS inhibitors (44.0%) |
| Hwang 2010  Retrospective cohort (125)  1983 - 2007  South Korea | Korean patients  Mean 36.5  (SD 11.4)  56.0% males | Mean 62.8 (SD 21.9)/  UP: Mean 3.10 g/d  (SD 1.90) | ACEi/ARB (100%), Prednisolone (20.0%), Prednisolone + cyclophosphamide (14.4%) |
| Jia 2022  Prospective cohort (140)  07/2008 - 12/2016  China | Chinese patients  Median 29.0  (IQR: 24.0, 38.0)  54.3% males | Median 82.0  (IQR: 67.1, 91.6)/  UP: Median 0.58 g/d  (IQR: 0.38, 0.78) | RASB (90.0%), Tonsillectomy (1.4%), Only use CS (14.3%), CS + immunosuppressants (48.6%), CS + CTX (30.0%), CS + MMF (15.7%), CS + LEF (2.9%) |
| Kee 2019  Retrospective cohort (33)  01/2010 - 12/2015  South Korea | Korean patients  Mean 49.3  (SD 11.6)  75.8% males | Mean 71.3 (SD 27.5)/  UPCR: Mean 3.15 g/g  (SD 2.52) | RASBs (75.8%) |
| Koike 2024  Retrospective cohort (991)  04/01/2005 - 08/31/2015  Japan | Japanese patients  Median 37.1  (IQR: 26.8, 50.4)  50.7% males | Mean 75.4 (SD 28.7)/  UP: Median 0.80 g/d  (IQR: 0.46, 1.41) | RASi (57.2%), CS0 (36.0%)/ CS1 (5.8%)/ CS2 (58.2%) |
| Le 2012  Retrospective cohort (1155)  1989 to 2005  China | Chinese patients  Mean 34.0  (SD 9.0)  49.7% males | Mean 89.0 (SD 33.0)/  UP: 0.89 g/d  (IQR: 0.51, 1.59) | NR |
| Moriyama 2014  Retrospective cohort (1012)  1974 - 2011  Japan | Japanese patients  Mean 32.9  (SD 12.0)  40.5% males | Mean 78.5 (SD 26.2)/  UP: Mean 1.19 g/d  (SD 1.61) | Corticosteroids (26.9%), Corticosteroid therapy combined with tonsillectomy (11.7%), Tonsillectomy alone (1.5%), Immunosuppressive agents such as cyclophosphamide, mizoribine, and rituximab (1.5%), Reninangiotensin-aldosterone system inhibitor (28.9%), A |
| Nam 2014  Retrospective cohort (500)  2002 - 2010  South Korea | Korean patients  Mean 37.1  (SD 12.0)  43.0% males | Mean 87.3 (SD 28.5)/  UP: Median 0.50 g/d  (IQR: 0.10, 1.50) | RAS blockers (78.0%), Corticosteroids (11.0%), Fish oil (4.0%) |
| Pitcher 2023  Retrospective cohort (2439)  2013 - 2020  UK | Caucasian patients  Mean 41.0  (SD 15.0)  71.0% males | Mean 55.0 (SD 29.0)/  UPCR: 2.42 g/g  (SD 3.57) | NR |
| Sarcina 2016  Retrospective analysis of three prospective, randomized clinical trials (325)  1989 - 2005  Italy, Switzerland | Caucasian patients  Mean 38.5  (SD 12.9)  0.745 | NR/  UP: Mean 2.35 g/d  (SD 1.50) | RAS blockers (50.5%) |
| Sevillano 2017  Retrospective cohort (112)  Not reported  Spain | Caucasian patients  Mean 41.5  (SD 17.9)  69.6% males | Mean 58.0 (SD 34.0)/  UP: Median 1.40 g/d  (IQR: 0.40, 2.70) | Corticosteroid monotherapy (27%), Corticosteroid + mycophenolate mofetil (61%), Corticosteroid + azathioprine (7%), Corticosteroid + cyclophosphamide (5%) |
| Shen 2025**  Retrospective cohort (2141)  01/2003 - 06/2023  China | Chinese patients  (100% males)  Median 34.0  (IQR: 28.0, 43.0)  51.0% males | Median 80.0  (IQR: 52.0, 103.0)/  UP: Median 1.26 g/d  (IQR: 0.65, 2.40) | RAS inhibitors (91%), Corticosteroids (43%), Immunosuppressant (26%), Hydroxychloroquine (27%) |
| Stamellou 2024  Prospective cohort (421)  2010 - 2012  Germany | Caucasian patients  Mean 51.6  (SD 13.5)  67.0% males | Mean 52.5 (SD 22.4)/  uACR: Median 0.40 g/g  (SD 0.10) | Antihypertensive therapy (96.4%), ACEi (59%), ARB (49.2%), Dihydropyridines( nifedipine-type) (35.7%), Diuretics (48.7%), Thiazides (24.7%), Loop diuretics (23%), Aldosterone antagonists (3.4%), Statins (44.6%), Immunosuppressives (6%), Glucocorticoids (1 |
| Stangou 2018  Retrospective cohort (457)  1990 - 2010  Greece | Greek patients  Mean 41.3  (SD 14.3)  66.3% males | Mean 64.1 (SD 30.7)/  UP: Mean 1.70 g/d  (SD 2.00) | Oral prednisolone (16.6%), methylprednisolone followed by oral prednisolone (12.5%), Oral prednisolone followed by azathioprine (7.0%), Oral prednisolone followed by MMF (2.0%), IV methylprednisolone pulse followed by prednisolone and IV cyclophosphamide/ |
| Takada 2019  Retrospective cohort (189)  03/1981 - 12/2013  Japan | Japanese patients  Mean 36.4  (SD 11.5)  50.8% males | Mean 75.2 (SD 23.1)/  NR | Anti-hypertension drugs (33.86%), Tonsillectomy (100%) |
| Tanaka 2013  Retrospective cohort (1400)  01/1982 - 12/2010  Japan | Japanese patients  Mean 36.1  (SD 15.4)  48.7% males | NR/ NR | NR |

*** Majority of patients.**

** Wakai et al 2006 and Tang et al 2024 were not listed here because Tang et al 2024 is a companion publication of Shen et al 2025 and Wakai et al 2006 is a companion publication of Goto et al. 2009. Abbreviations: % = percentage; ACEi = angiotensin converting enzyme inhibitors; ARB = angiotensin II receptor blocker; BB = beta-blockers; CCB = calcium channel blockers; CS = corticosteroids; CTX = cyclophosphamide,; eGFR = estimated Glomerular Filtration Rate; IQR = interquartile range; IV = intravenous; LEF = leflunomide; MMF = mycophenolate mofetil; NR = not reported; RAAS = renin-angiotensin-aldosterone system; RAS = renin-angiotensin system; RASB = renin-angiotensin system blockers; RASi = renin-angiotensin system inhibitors; SD = standard deviation; uACR = urine albumin-to-creatinine ratio; UP = urinary protein; uPCR = urine protein-to-creatinine ratio.

Supplemental Table 6. Risk ratio of renal failure based on baseline proteinuria

| Study | Definition of renal demise | Proteinuria g/d | | | |
| --- | --- | --- | --- | --- | --- |
|  |  | ≤ 0.30 | 0.31 – 0.99 | ≤ 0.50 | 0.51 – 1.00 |
| Nam 2014 | dialysis, transplantation | Ref | RR 1.00  (95% CI - not estimable) |  |  |
| Moriyama 2014 | dialysis, transplantation |  |  | Ref | RR 1.13  (95% CI - not calculated) |
| Wakai 2006 | dialysis | Ref | RR 2.97  (95% CI 0.86 to 10.30) |  |  |
| Chen 2018 | eGFR < 15*, transplantation |  |  | Ref | RR 1.00  (95% CI - not estimable) |
| Hwang 2010 | eGFR < 15*], KRT | Ref | RR 10.01  (95% CI 1.25 to 80.25) |  |  |
| Kee 2019 | eGFR decline >30% | Ref | RR 0.21  (95% CI 0.04 to 0.88) |  |  |
| Sarcina 2016 | ESKD [no further definition provided] | Ref | RR 1.03  (95% CI 0.99 to 1.07)​ |  |  |
|  | SCr doubling | Ref | RR 1.03  (95% CI 1.00 to 1.07) |  |  |

Abbreviations: Ref = reference; CI = confidence intervals; RR = risk ratio; SCr = serum creatinine.

Note: Chen 2018 and Nam 2018 reported zero events in both arms. A small continuity correction (e.g., adding 0.5 events to each cell) was used to calculate the RR. Moriyama 2014 reported incidence rates with no measure of variance. As such, CIs could not be calculated.

Supplemental Table 7. Quality assessment according to the Newcastle–Ottawa Scale

| **Study, Year** | Total Score |
| --- | --- |
| Ai 2020 | 6 |
| Chen 2018 | 6 |
| Faucon 2025 | 7 |
| Goto 2009 | 5 |
| Gutiérrez 2012 | 6 |
| Hirano 2013 | 7 |
| Hwang 2020 | 7 |
| Jia 2022 | 7 |
| Kee 2019 | 5 |
| Koike 2024 | 7 |
| Le 2012 | 7 |
| Moriyama 2014 | 7 |
| Nam 2014 | 7 |
| Pitcher 2023 | 7 |
| Sevillano 2017 | 7 |
| Shen 2025 | 7 |
| Stamellou 2024 | 7 |
| Stangou 2018 | 7 |
| Takada 2019 | 8 |
| Tanaka 2013 | 5 |

Note: Three of the 23 included references were not assessed due to the following reasons: Sarcina et al 2016 is a retrospective analysis of three prospective, randomized clinical trials; Tang et al 2024 is a companion publication of Shen et al 2025 and Wakai et al 2006 is a companion publication of Goto et al 200
